# Parry Romberg Syndrome: A Case Report and an Insight Into Etiology

**DOI:** 10.7759/cureus.41465

**Published:** 2023-07-06

**Authors:** Munish Kumar, Robin Singla, Gagandeep Singh, Rishabh Kasrija, Manish Sharma

**Affiliations:** 1 Department of Oral Surgery, Guru Nanak Dev Dental College, Sunam, IND; 2 Department of Oral Surgery, JSS Dental College, Mysuru, IND; 3 Department of Oral Pathology and Microbiology, JMF's (Jawahar Medical Foundation's) ACPM (Annasaheb Chudaman Patil Memorial Medical) Dental College, Dhule, IND

**Keywords:** neurological diseases, ­trauma, cbct, parry romberg syndrome, hemifacial atrophy

## Abstract

Parry-Romberg syndrome (PRS) is a rare degenerative disorder of unknown cause that causes slow, progressive atrophy on one side of the face. The cause may be a malfunction of the sympathetic nervous system, with or without neurological symptoms. Atrophy usually begins in childhood and progresses gradually over several years. Stabilization can take up to 20 years. There is no definitive cure for this condition, but once the condition is stabilized, reconstructive surgery of the damaged skin and soft tissue can correct the deformity. The objective of this article is to present an insight into the etiology of PRS with a case report of a 15-year-old male patient, who was diagnosed with PRS due to trauma and developed progressive hemifacial atrophy without neurological manifestations. PRS is a progressive disease that severely affects one side of the face. Because of its devastating effects on the entire body, treatment requires a multidisciplinary approach. Further research is needed to clearly understand the etiology and provide patients with accurate treatment plans.

## Introduction

Parry-Romberg syndrome (PRS), also known as progressive hemifacial atrophy, was initially described by CH Parry and MH Romberg in 1825 and 1846, respectively [1]. It is a very rare disorder that occasionally affects the left side of the face, initially involving the skin and subcutaneous tissues, gradually progressing to the muscles and underlying osseous structures, with or without neurological, cardiac, ophthalmic, and endocrine involvement [2].

Regarding the pathophysiology of this condition, many theories have emerged over time and possible suggested factors that may contribute to its pathogenesis include trauma, viral infections, sympathetic nervous system dysfunction, genetic factors, and autoimmunity; however, the exact etiology is currently unknown [3,4].

PRS affects females more than males and does not have a definitive cure. A multidisciplinary approach with a team of medical professionals, consisting of plastic surgeons, psychologists, audiologists, surgeons, and dental specialists, is required to improve the patient's appearance. Reconstructive surgeries along with immunosuppressive drugs have been reported to provide considerable results in the treatment of patients [5]. In this paper, we report the unique case of a 15-year-old male patient diagnosed with Parry-Romberg syndrome with a history of trauma two years back and no associated neurological, cardiac, ophthalmic, or endocrine symptoms. A short insight into its etiology has also been provided.

## Case presentation

A 15-year-old male was reported to the Department of Oral and Maxillofacial Surgery with the chief complaint of facial asymmetry and scarring on the right side of the middle one-third of the face in the past year. Written informed consent was obtained from the patient for the use of his medical records and photographs for this report.

History and general examination

The history of the patient revealed that the patient had trauma while playing kabaddi two years back, at the age of 13 years, and since then, the patient had slow “shrinking” of the right side of the face, which was noted by his family members. Before that, the patient's face was normal, as confirmed by photographs. He had no relevant medical, dental, or family history.

Upon clinical examination, the patient presented with normal vital signs and systemic conditions. He was alert and healthy with no signs of mental or psychological instability. No speech or hearing impairments were observed.

Extraoral examination

**Figure 1:**
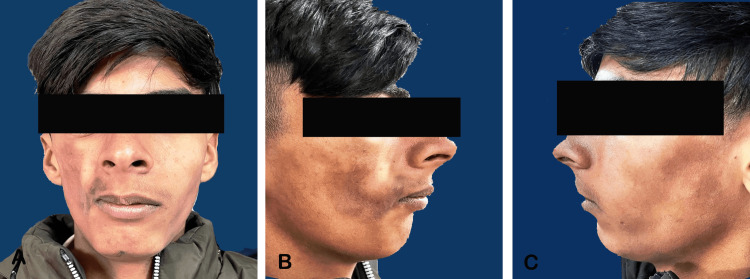
Extraoral features related to Parry Romberg syndrome A. Front profile; B. Left side profile; C. Right side profile

The face was asymmetric due to a right-sided deformity. There was a noticeable inter-pupillary cant, with the lowering of the right eye as compared to the left eye. There was significant atrophy and a scar-like defect (coup de saber) at the level of the right maxillary and zygomatic regions compared with the other side. Atrophy was observed at the level of the eyes, malar region, lips, and mandible on the right side of the face, leading to the deviation of the nose and chin to the right side. The right half of the upper and lower lips exhibited contracture along with a slight commissural lift. The ears were symmetrical and normal (Figure 1).

Intraoral examination

**Figure 2:**
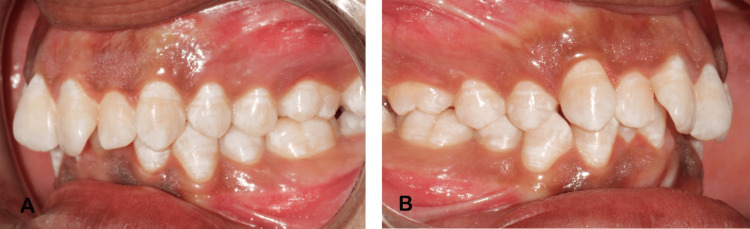
Intraoral features A. Permanent dentition; B. Class 1 molar relation

Intraoral examination revealed a permanent dentition stage with a class I molar relationship, deep bite, and normal occlusion (Figure 2). No soft tissue abnormalities were observed. The size, position, and movement of the tongue showed no abnormalities. Mouth opening was within the normal range (45 mm).

Provisional diagnosis and tests

**Figure 3:**
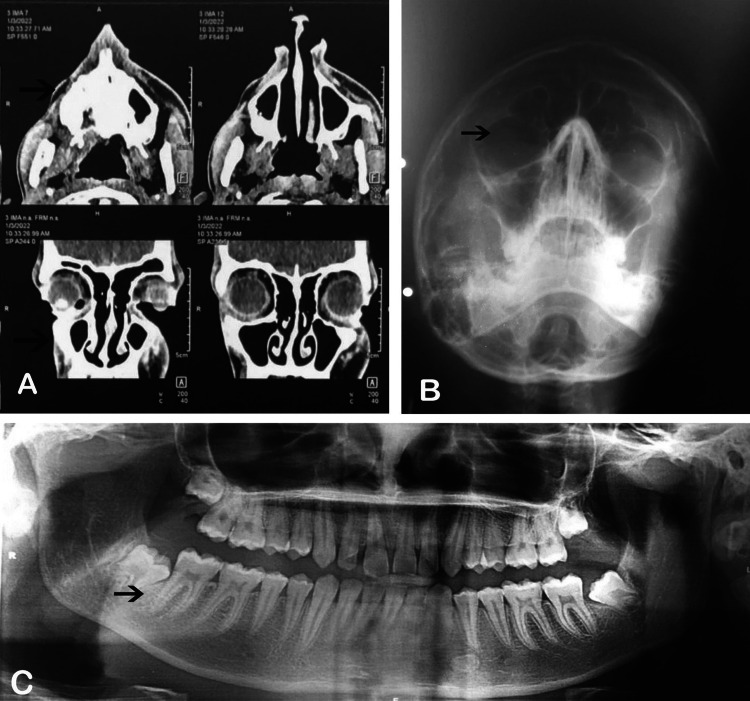
Radiographic features A. CT scan image showing atrophied facial muscles; B. Smaller frontal and maxillary sinus; C. Normal root development in dentition

A preliminary diagnosis of hemifacial atrophy was made prior to the investigations, and routine blood tests, radiographs, and CT scans were performed. Radiographic examination revealed no significant findings on orthopantomogram (OPG). All teeth showed normal root development in the presence of developing third molars. X-ray examination of the paranasal sinus (PNS) showed smaller frontal and maxillary sinuses on the right side than on the left side (Figure 3).

**Figure 4:**
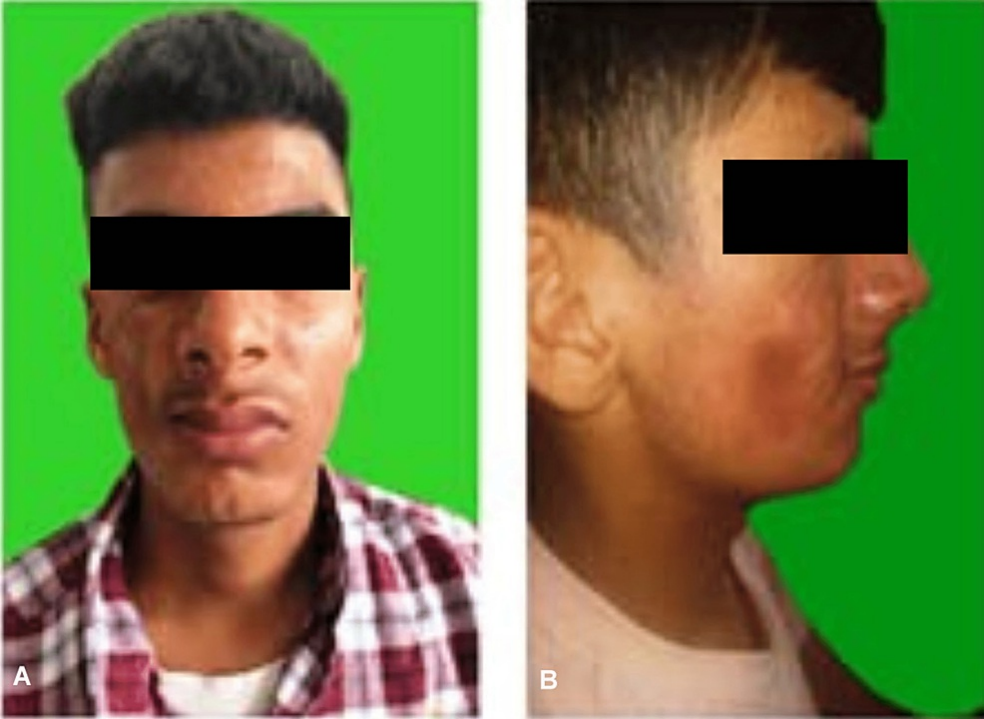
Muscle atrophy on the right side of the face A. Front view; B. Side view

A non-contrast CT scan of the paranasal sinuses revealed atrophied skin, subcutaneous tissues, and facial muscles overlying the right maxillary bone. Osseous destruction was not observed. Muscle atrophy was observed on the right side and the middle one-third of the face (Figure 4).

All laboratory tests, including serum alkaline phosphatase (ALP), serum calcium, serum sodium, serum potassium, and complete blood count showed no remarkable findings except for serum vitamin D levels, which were less than normal. Anti-nuclear antibody titers were absent.

Diagnosis and treatment

Based on clinical, radiological, and hematological findings, the patient was diagnosed with PRS. Linear type of scleroderma is a prime differential diagnosis for PRS. "Coup de sabre," a cutaneous characteristic is present on the forehead in scleroderma, but it doesn't show subcutaneous muscle atrophy. Another differentiation is anti-nuclear antibody titers, present in scleroderma. Since the patient was healthy with no neurological or dental manifestations, supportive treatment was provided, with follow-up until growth cessation and stabilization of the disease. He was treated with tablet methotrexate 10 mg OD weekly for two months along with tab prednisolone 20 mg BD prescribed in a tapered dose for two months. The topical use of calcipotriol betamethasone ointment was prescribed to apply twice daily for two months. Vitamin D 60000 IU weekly was given for four weeks.

Follow-up after two months

The patient did not show any progression in symptoms or neurological manifestations. The patient was kept on future follow-up to check for stabilization of the disease process and completion before further planning of reconstructive surgeries for facial defects.

## Discussion

PRS is an uncommon neurocutaneous disorder that mainly affects the left side of the face, leading to hemifacial atrophy [6]. Frequently, females are more affected than males [6,7]. It affects the underlying tissue layers with or without neurological involvement. According to the classification by Guerrerosantos et al., who classified this syndrome into four types depending on the involvement of underlying tissues, our patient was categorized as Type 1 or Type 2 (mild involvement of the soft tissues of the face and the skin) [7].

This syndrome usually starts in the first two decades of life, usually before the age of 15 years, and reaches a maximum until the age of 25 years [8], as was the case with our patient, whose symptoms started at the age of 13 years after facial trauma. Our patient presented with no neurological signs and symptoms, normal dentition, or tongue. No abnormalities in ocular vision or ears were noticed, which is in contrast to previous case reports [9,10]. The patient presented with significant atrophy of the right malar prominence, zygomatic process, and maxilla [11]. The paranasal sinuses were also affected.

Because there is no definitive cure for the disease to slow or prevent deterioration, individualized treatment plans are required for every case [1]. Our patient was in the growth phase and had no neurological findings. Hence, we decided to provide supportive care to the patient and perform regular follow-ups to look for stabilization of the disease process and completion of growth. The patient did not show any deterioration after a two-year follow period. Facial reconstructive surgeries using grafts are planned after cessation of growth.

Insight into etiology

Various theories have been proposed to explain the etiopathogenesis of PRS, including vascular, genetic, autoimmune, neurogenic, and exogenous traumas [2,3]. The preliminary hypothesis for the etiology of PRS is trophoneurosis. The dysfunction of trophic fibers of the trigeminal and peripheral nerves leads to ophthalmic symptoms like enophthalmos and hemifacial atrophy along with bone atrophy on the ipsilateral side. Another possible etiology can be dysregulation of neural crest cell migration. It shows symptoms of intracranial aneurysms along with orbital neuromas, odontogenic fibromas, and hamartomas [12,13].

Intracranial vascular lesions and vascular lesions along the course of the trigeminal nerve show lymphocytic vasculitis (a feature seen in Rasmussen encephalitis) on histopathological examination, suggesting a neurovasculitis theory [14]. The autoimmune process is the most commonly predicted etiology in PRS, as features overlap with systemic sclerosis or scleroderma and findings coexist with the presence of autoantibodies. Some authors consider it a variant of linear scleroderma because of its similar histopathology [15]. Jenny et al. detected differentially expressed genes in PRS patients' diseased tissues compared to normal subjects. The results showed that there were 349 upregulated genes and 111 down-regulated genes in PRS patients. Upregulated genes, such as IL24, including GFCSF3, ADAMTS4, and nuclear factor-κB, are strongly associated with inflammation, cell proliferation, immune responses, and IL24 [16].

Viral and bacterial infections are also suggestive of PRS etiology. Primarily slow viruses, such as herpes and Borrelia burgdorferi, are causative organisms. Metabolic disorders cause lipodystrophy and facial atrophy due to the subcutaneous dissolution of fat tissues. Traumatic insults from accidental, operative, or surgical procedures may lead to PRS. It was hypothesized 24%-34% of patients with PRS have a history of trauma [17]. Although PRS has been known for more than 150 years, the specific etiology and mechanism of the disease are poorly understood, and the treatments mainly focus on reconstructive surgeries of the atrophic site. Future studies are required for a clear understanding of the etiology of PRS and for designing a specific treatment plan for each patient.

Management

Parry Romberg is a self-restraining disease. There is no definite treatment available for PRS. The symptomatic medication provides transient relief. Efforts have been made in various clinical studies to rectify the deformities and prevent the advancement of the disease [7,9]. Most of the cases opt for therapy to treat coexisting autoimmune disorders, which merely affect the sustainability of distorted tissues [7]. The use of methotrexate drug is a standard therapy for progressive disease along with oral prednisolone 1 mg/day for three months in a tapering dose at the end. Reconstructive surgeries for facial tissue dystrophy with fat and cartilage grafts are also recommended [18]. We have used a noninvasive approach, as the patient is on follow-up for stabilization of the disease process before planning surgical intervention.

## Conclusions

As PRS is mild in its early stages, it is often overlooked or misdiagnosed during the routine visits of the patient. An accurate and early diagnosis of PRS requires that physicians involved in the initial patient examination and treatment have a thorough knowledge of the condition. A detailed examination of the patient's medical history, previous photographs, and comprehensive physical examination is required to identify signs of progressive loss of skin, fat, muscle, and bone. Patients with asymmetric faces may experience psychological problems. Therefore, clinical psychologists should be involved in treatment at a very early stage.

In this case, we used a noninvasive approach, as the patient is on follow-up for stabilization of the disease process before planning surgical intervention. The patient was recommended for an autogenous fat graft for soft tissue asymmetry in the future. This case report along with the etiology documents the classic features and hence contributes to the understanding of the disease.
